# Side effects of braces; a cross-sectional survey

**DOI:** 10.1186/1748-7161-8-S1-O45

**Published:** 2013-06-03

**Authors:** M Tavernaro, F Tessadri, A Zonta, S Negrini

**Affiliations:** 1 ISICO (Italian Scientific Spine Institute), Milan, Italy; 2 Orthotecnica, Trento, Italy; 3 University of Brescia, Brescia, Italy; 4 IRCCS Don Gnocchi, Milan, Italy

## Background

Any therapeutic intervention causes side effects, that should be taken into account when prescribing it, and explained to patients and families to reach an informed consent to treatment. In the field of bracing, only psychological impact has been considered and, until now, as far as we know, nobody presented results on the bodily consequences of brace wearing.

## Aim

To check through a questionnaire the bodily side effects of bracing.

## Methods

Design: cross-sectional survey. Methods: a specific questionnaire has been developed and validated to evaluate the following domains: skin, respiration, mobility, Activities of Daily Life. The questionnaire was sent by emails to 1551 idiopathic scoliosis or hyperkyphosis patients of age between 10 and 18, including also patients not braced to have a control group; response rate was 11.0%. Population: 170 patients, 75.3% females, age 14.4±2.0; 30 controls (CG) and 140 braced (BG) for 1.1 years, either with SpineCor (BG-S n=14) or rigid orthosis (BG-R n=126); the last group was also divided into TLSO (BG-T n=104) and LSO (BG-L n=22). We compared each answer and the average indexes of all domains.

## Results

The median of answers was never (100%) in CG; “sometimes” (13%), “almost never” (29%); and “never” (58%) in BG, where the answer “always” reached a maximum of 22.1% in one question (minimum 0.7%). BG and all BG subgroups had almost all answers, and all indexes, statistically significantly higher than CG. Conversely, there were almost no differences between the different BG subgroups: BG-S and BG-L had low samples, and some tendencies to differences appeared. In comparison, BG-S had problems in toileting, while BG-R with sweat.

## Conclusions

With a cross-sectional study, it is not possible to generalize too much about the results, since presumably only most interested patients answered, not well representing the entire population of braced patients. Nevertheless, with this survey it is possible to gather some data on the main domains to be explored, and on some differences among sub-groups. It is possible to conclude that there are bodily low-degree (maximum “sometimes”) side-effects of braces, with specific differences according to the brace used; moreover, no brace is without side-effect.
